# A Complicated Clinical Course of Community-Acquired Staphylococcus aureus Infective Endocarditis

**DOI:** 10.7759/cureus.35973

**Published:** 2023-03-10

**Authors:** Shannay E Bellamy, William Ott, Josh D Kolb, Khurram Malik

**Affiliations:** 1 Internal Medicine, Jersey City Medical Center, Jersey City, USA; 2 Infectious Diseases, Jersey City Medical Center, Jersey City, USA

**Keywords:** septic embolic stroke, neurologic complication, native valve, staphylococcus aureus endocarditis, methicillin-sensitive staphylococcus aureus, infective endocarditis

## Abstract

Infective endocarditis (IE) is an infection of the heart's endocardial surface, heart valves, or implanted cardiac devices, with the most common causative organism being *Staphylococcus aureus*. The clinical presentation of IE can be variable, with some patients presenting with multisystemic complications, including renal, pulmonary, cutaneous, and neurologic complications. Cerebral infarction is the most common complication of IE. Here we present a case of a young male with *S. aureus* IE of a native cardiac valve who developed multiple complications during his clinical course.

## Introduction

Infective endocarditis (IE) is a life-threatening inflammatory condition of the endocardium and heart valves with a mortality of up to 30% in hospitalized patients [1]. *Staphylococcus aureus* has been identified as the most common cause of IE and *S. aureus* infective endocarditis (SAIE) is associated with significant morbidity and mortality [2]. When SAIE occurs in patients without a history of hospitalization or recent vascular or surgical procedure, it is known as community-acquired SAIE. In these instances, SAIE is the result of bacteremia caused by methicillin-sensitive *S. aureus* (MSSA), and the source of the bacteremia is commonly not identified [3]. Risk factors for community-acquired SAIE include a history of rheumatic heart disease, intravenous drug use, immunosuppressive therapy, and dental caries [4]. Regardless of the causative micro-organism, the management of IE involves medical therapy and, often, surgical interventions. The optimal timing of surgical intervention is controversial and often influenced by complications of IE [5]. We present a case of a young male without a significant medical history who was diagnosed with SAIE and later developed life-threatening complications.

## Case presentation

A 35-year-old male with a medical history significant for anxiety and depression presented to the emergency department (ED) with a one-day history of altered mental status. He was noticed by his wife to be confused, unable to recognize her, and responding to questions inappropriately, with evidence of bladder and bowel incontinence. She denied any witnessed seizure activity and any history of illicit drug use. He had been experiencing fever, chills, myalgias, headache, nausea, and a rash on the palms of the hands for the prior 10 days, which had caused him to return home early from a business trip to South Florida. Six days before this presentation, he was seen in the ED for these symptoms, where he was diagnosed with hand, foot, and mouth disease and discharged home with recommendations for supportive treatment with oral hydration and analgesia. No laboratory investigations were done at this time.

**Figure 1:**
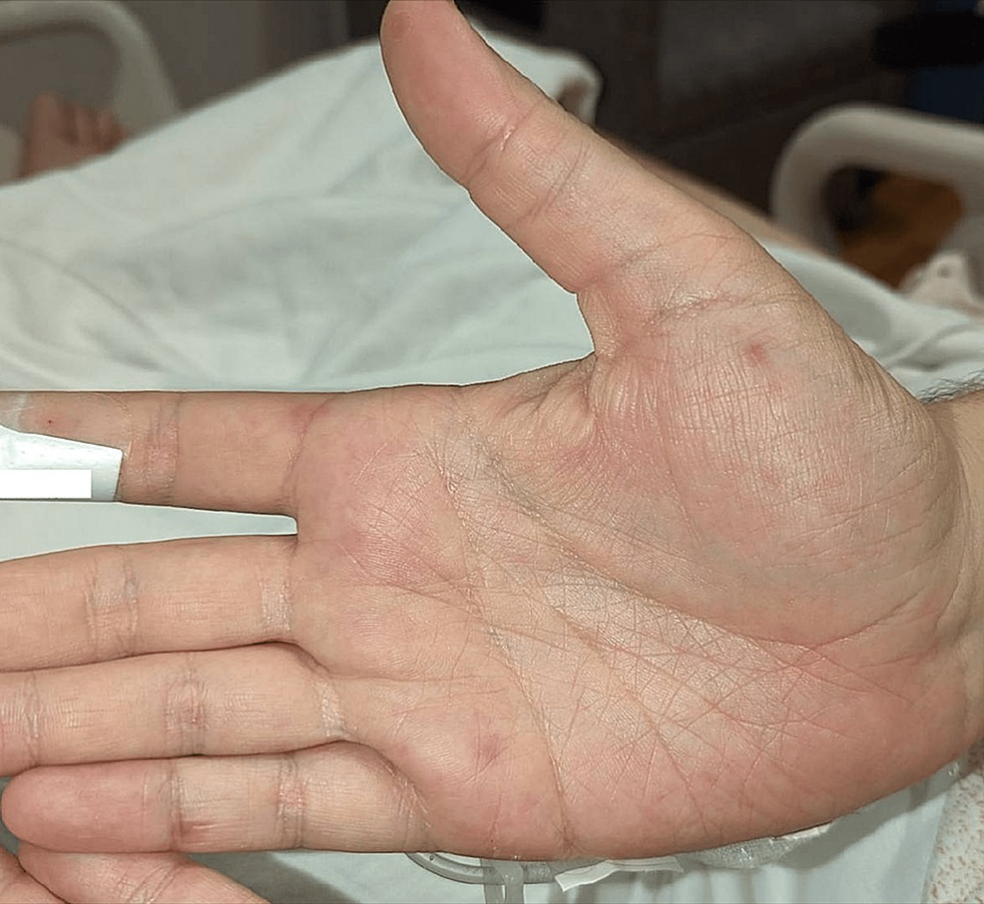
Macular rash on the palms of the hands (Janeway lesions)

**Figure 2:**
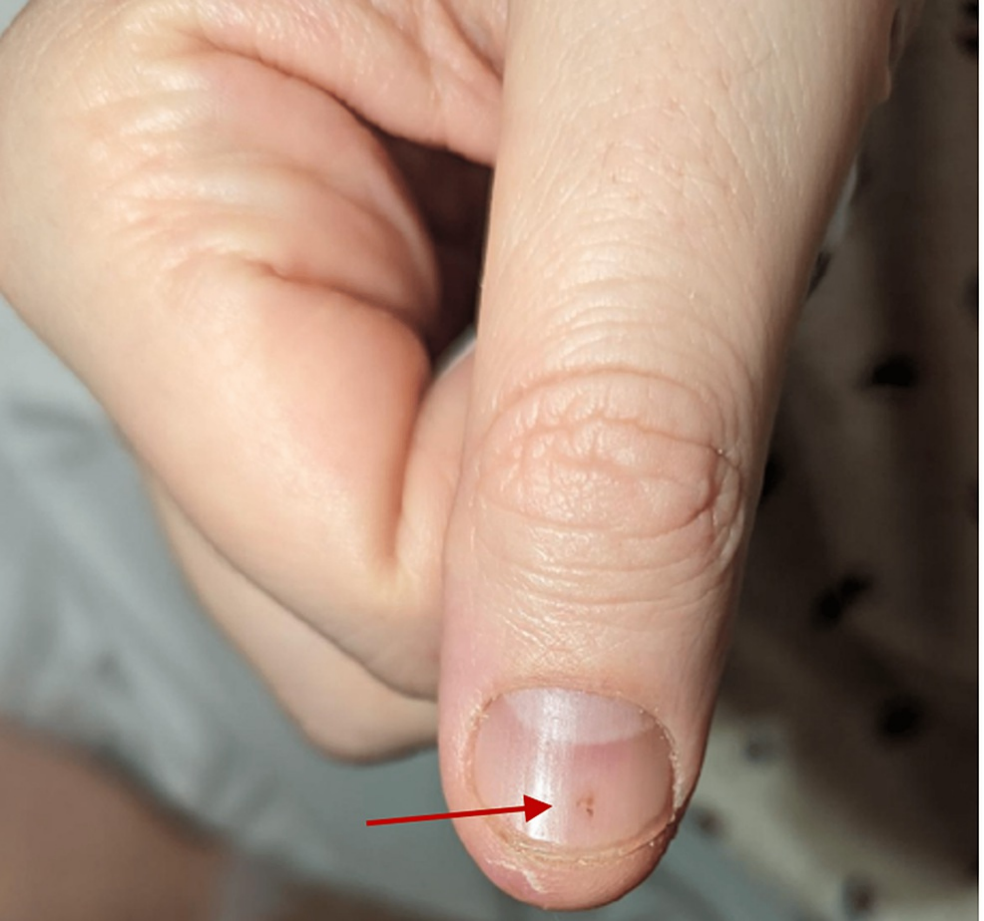
Splinter hemorrhage (red arrow) on the nail of the thumb

On presentation, he was normotensive, tachycardic with a heart rate of 127 bpm, febrile at 104.1°F, and tachypneic at 30 breaths per minute on oxygen supplementation by a non-rebreather mask. On physical examination, he was found to be disoriented and drowsy but arousable, opening his eyes to voice and moving all extremities equally and spontaneously without any focal deficits. There were no neck rigidity and Kernig’s and Brudzinski’s signs. Physical examination was otherwise significant for scleral icterus and diffuse 2- to 3-mm erythematous maculae on bilateral hands and lower extremities, which were not blanchable, and splinter hemorrhage on the thumb (Figures 1, 2, respectively).

Fundoscopy was attempted; however, due to the patient's altered mental status and drowsiness, he was unable to keep his eyes open for a full assessment, and the exam was therefore unsuccessful. Cardiovascular, respiratory, and gastrointestinal examinations were otherwise unremarkable.

Initial laboratory investigations were significant for leukocytosis with white blood cells at 16,700 cells/uL with a neutrophil count of 13,694 cells/uL, normocytic anemia with hemoglobin 12 g/dL, thrombocytopenia with platelet count 60,000/uL, hyponatremia at 124 mmol/L, mild renal impairment with the blood urea nitrogen (BUN) level at 16 mg/dL, creatinine level 1.36 mg/dL, hyperbilirubinemia with bilirubin 7.0 mg/dL and elevated liver transaminases with alanine aminotransferase (AST) level at 102 units/L and aspartate aminotransferase level at 97 units/L. The lactic acid level was normal. Erythrocyte sedimentation rate (ESR), C-reactive protein (CRP), and procalcitonin levels were significantly elevated (Table 1).

**Table 1:** Laboratory investigations on presentation (H) indicates a value above the upper reference limit. (L) indicates a value below the lower reference limit.

Laboratory test on admission	Result	Normal range
Hemoglobin (g/dL)	12.0 (L)	14-18
Mean corpuscular volume (fL)	84	80.0-100.0
Platelets (cells/uL)	60,000 (L)	130,000-430,000
White blood cells (cells/uL)	16,700	4500-10,000
Prothrombin time (sec)	16.7	12-15.1
International normalized ratio	1.43	0.85-1.14
Partial thromboplastin time (sec)	27.5	25.4-36.7
Fibrinogen (mg/dL)	488	244-550
Sodium (mmol/L)	124 (L)	136-145
Potassium (mmol/L)	3.6	3.5-5.1
Chloride (mmol/L)	90 (L)	98-107
Serum bicarbonate (mmol/L)	26	20-31
Blood urea nitrogen (mg/dL)	26 (H)	9-23
Creatinine (mg/dL)	1.36 (H)	0.70-1.30
Calcium (mg/dL)	7.8	8.7-10.4
Aspartate aminotransferase (units/L)	97 (H)	8-34
Alanine aminotransferase (units/L)	102 (H)	10-49
Alkaline phosphatase (units/L)	267 (H)	46-116
Total bilirubin (mg/dL)	7.0 (H)	0.3-1.2
Haptoglobin (mg/dL)	388	43-212
Lactic acid (mmol/L)	1.70	0.50-1.99
Erythrocyte sedimentation rate (mm/hr)	78 (H)	0-15
C-reactive protein (mg/dL)	28.3 (H)	0-1
Procalcitonin (ng/mL)	61.48 (H)	<0.05

Ethanol and acetaminophen levels were within normal limits, and the urine drug screen was negative. Human immunodeficiency virus (HIV), hepatitis B and C viral serology, and rapid plasmin regain (RPR) were negative. Urinalysis was positive for blood and trace leucocyte esterase but negative for nitrites. Urine microscopy showed 5-10 white blood cells per high-power field (hpf), 5-10 red blood cells per hpf, and moderate bacteria, but was negative for casts. The upper respiratory viral panel was positive for coronavirus HKU1. A computed tomography (CT) scan of the head showed no acute hemorrhages or infarcts and was reported as a normal study.

The patient was assessed as having severe sepsis. Thrombotic thombocytocytopenic purpura (TTP) was thought to be a differential diagnosis on presentation due to clinical and laboratory features of altered mental status, fever, mild normocytic anemia and thrombocytopenia. Other differentials included (1) encephalitis versus menogoencephalitis due to the presentation of altered mental status in the patient with fever; (2) infective endocarditis due to the prolonged presentation of fever, non-specific symptoms of chills, myalgias, headache, and nausea with macular rash in palms in keeping with possible Janeway lesions and splinter hemorrhage; (3) malaria, thought to be less likely given no history of travel to endemic areas.

Blood and urine cultures were taken and he was treated with intravenous fluids and started on antimicrobial agents as per our sepsis protocol. Empirical antimicrobial therapy targeting the potential sources of sepsis included vancomycin, ceftriaxone, doxycycline, and acyclovir. A peripheral blood smear was done to further investigate the differential of TTP, but was negative for schistocytes; further investigation of the hyperbilirubinemia revealed a direct hyperbilirubinemia with direct bilirubin at 5.0 mg/dL. Thick and thin smears for malaria were negative. A transthoracic echocardiogram (TTE) was ordered and a lumbar puncture was planned.

**Figure 3:**
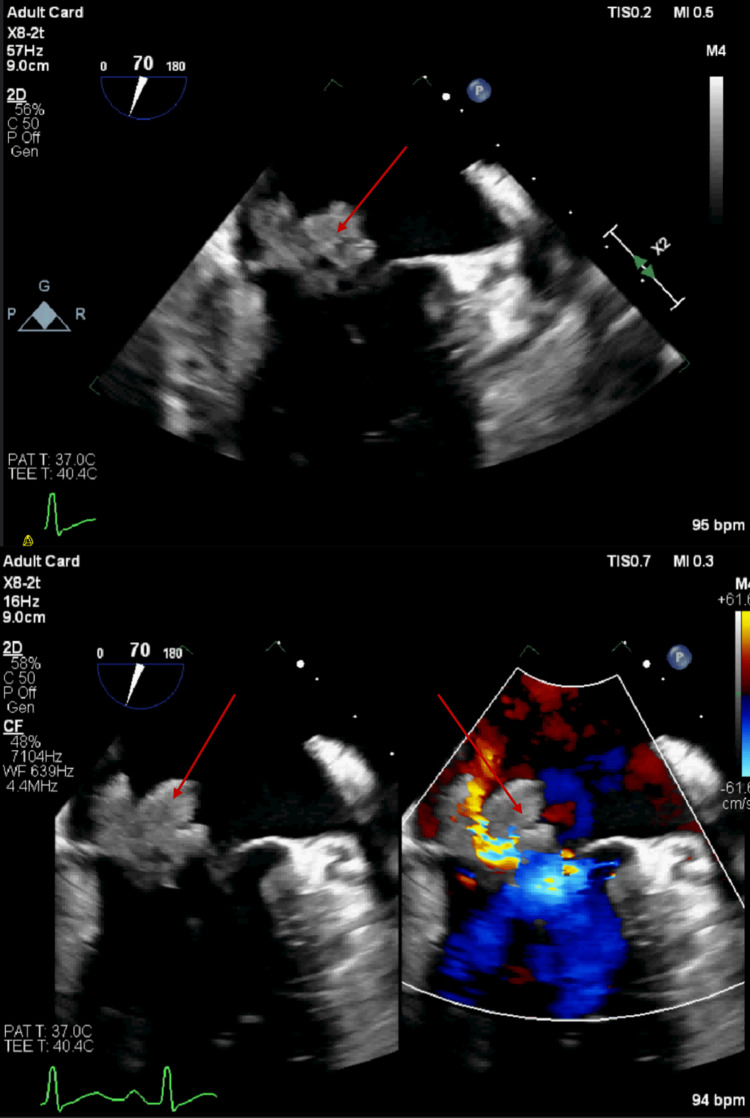
A transesophageal echocardiogram showing large vegetations on both the anterior and posterior leaflets Red arrows indicate valvular vegetation

He was admitted to the intensive care unit (ICU) for further monitoring. The following morning, the patient showed some clinical improvement in his mental status. While awaiting TTE, preliminary results of blood and urine cultures, taken on admission, were reported to be positive for the *Staphylococcus* species. As a result acyclovir, ceftriaxone, and doxycycline were discontinued. Vancomycin was continued pending final blood culture results. TTE done illustrated mild to moderate mitral valve thickening and mild mitral valve prolapse; however, IE could not be ruled out. The cardiology team was consulted and transesophageal echocardiography (TEE) was planned for the following day (day 2 of admission), for further evaluation of the mitral valve. TEE ultimately showed a severely thickened mitral valve with independently mobile, large vegetations on both the anterior and posterior leaflets measuring >1 cm as well as mild, posteriorly directed mitral regurgitation-findings consistent with infective endocarditis (Figure 3).

**Figure 4:**
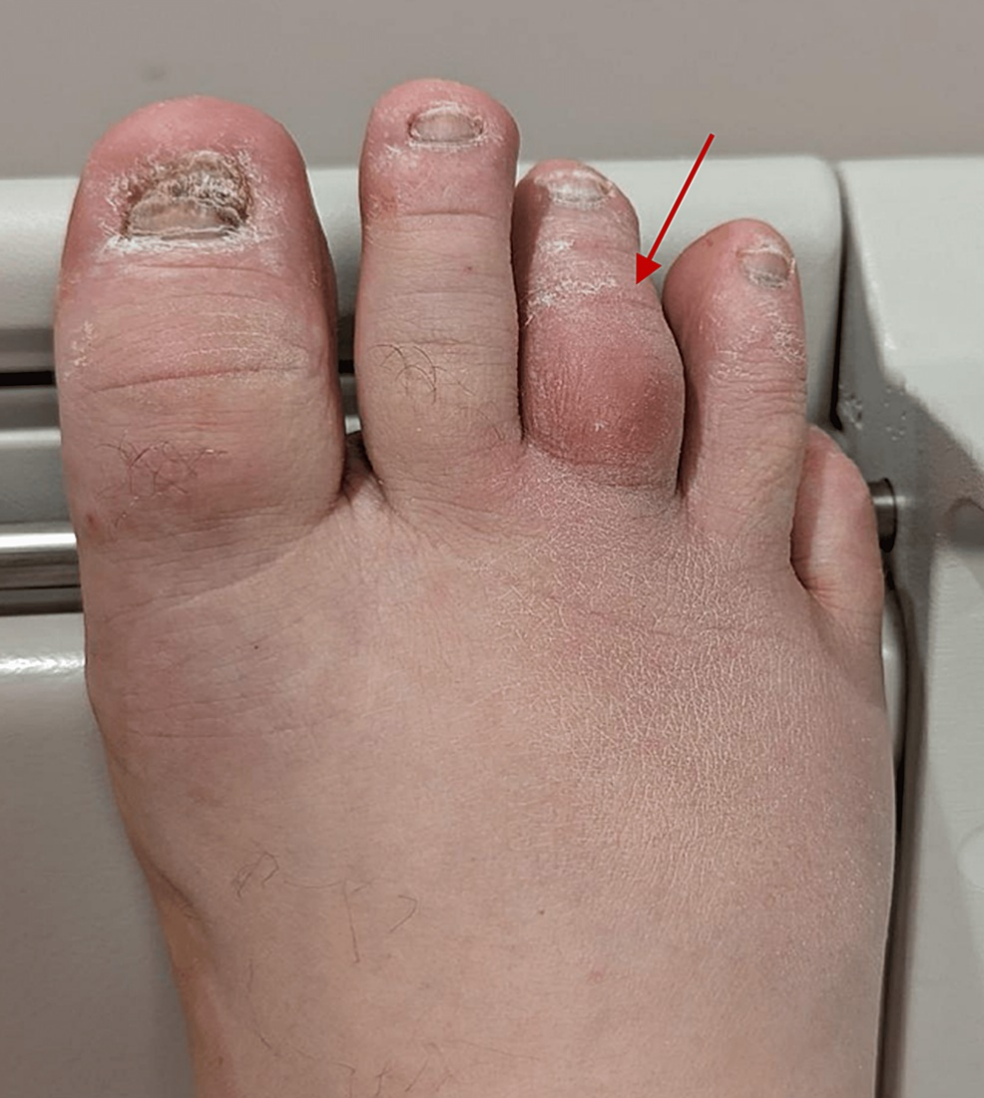
An image showing red swollen third toe, which occurred during transesophageal echocardiography

During TEE, the patient reported pain in his right third toe, which became red, painful, and swollen. This new clinical finding was likely due to digital ischemia caused by septic emboli occluding the microvasculature supplying that digit (Figure 4).

Subsequently, sensitivity reports identified the type of *Staphylococcus* species as methicillin-sensitive *S. aureus*, and antibiotic coverage was de-escalated from vancomycin to cefazolin. Further history revealed no recent hospitalizations, surgical procedures, lacerations, or abrasions, and he denied any intravenous drug use. A close physical examination did not reveal obvious trauma, lacerations, or evidence of cellulitis.

Despite appropriate antibiotic therapy with cefazolin, the patient continued to have intermittent fevers, and antibiotic therapy was changed to nafcillin. Due to the size of the valvular vegetation and the risk for more significant and possibly life-threatening potential embolic events, cardiothoracic surgery was consulted for early mitral valve replacement.

**Figure 5:**
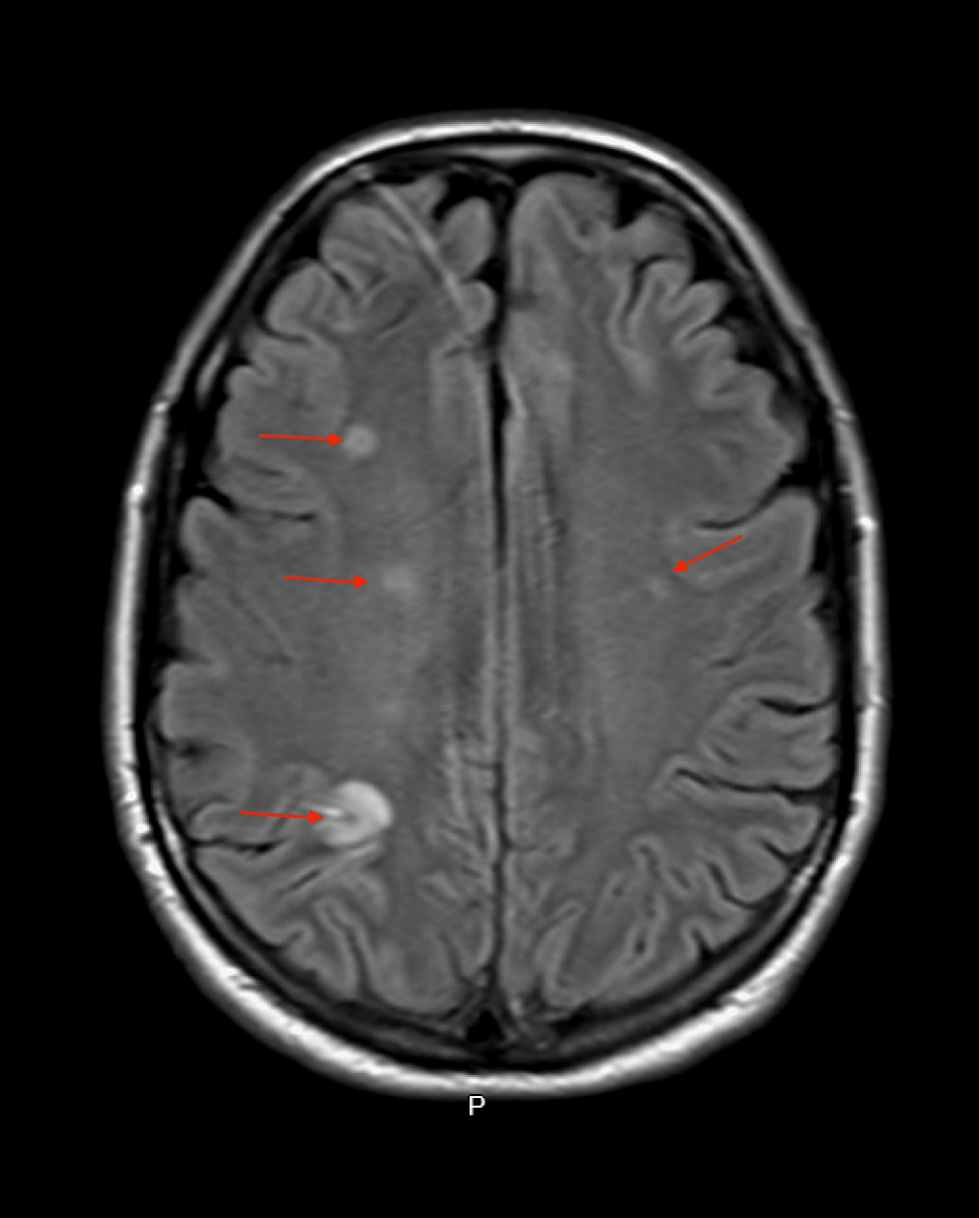
Magnetic resonance imaging of the brain showing multiple cerebral infarcts (red arrows)

On day 3 of admission, approximately two hours prior to his scheduled surgery, the patient complained of weakness in the left arm, with a physical examination significant for mild left hemiparesis. Urgent magnetic resonance imaging (MRI) of the brain demonstrated multiple bilateral cerebral infarcts, including subacute cortical infarcts to the right parietal cortex, subcortical right frontal lobe infarcts, left frontal lobe cortical infarcts, right parietal deep white matter infarcts, left inferior occipital lobe cortical infarcts and infarcts to the cerebellar vermis and left cerebellar hemisphere. The largest infarct involved the right parietal lobe with small areas of susceptibility artifact consistent with petechial hemorrhage (Figure 5).

With these new neurological and imaging findings, a multidisciplinary discussion involving cardiology, cardiothoracic surgery, and neurology teams, the patient, and his family concluded that the risks of surgery outweighed the benefits at that time, and surgery was deferred for two weeks. At the patient's request, he was transferred to another hospital for surgical intervention. Attempts made to follow up on the patient's course after the transfer to an out-of-state hospital were unsuccessful.

## Discussion

The prognosis of SAIE depends on timely diagnosis and management [6]. The findings of a new cardiac murmur in a patient with a fever highly suggest IE. This combination of findings, however, is observed in only about 50% of patients with IE. The clinical presentation of IE is variable, with some patients presenting with non-specific symptoms such as fever, malaise, changes in appetite or weight, and others presenting with clinical manifestations due to septic embolic complications such as respiratory, neurological, or cutaneous manifestations [7]. Specific cutaneous manifestations of IE such as splinter hemorrhages, Osler’s nodes, and Janeway lesions, are seen in less than 10% of cases but, when observed, can strengthen the diagnostic probability [4]. Cutaneous manifestations of IE are often associated with a more complicated course of IE and a worse prognosis [8].

The case we describe here initially presented with a constellation of non-specific symptoms in addition to a "rash" with no detection of a murmur on clinical examination. In the presence of these non-specific symptoms, his rash was not recognized as Janeway lesions, one of the uncommon cutaneous manifestations of IE. He presented again six days later with fever and altered mental status, meeting the criteria of severe sepsis. Again, the rash was not immediately recognized as Janeway lesions, but rather a differential diagnosis of possible petechiae in the setting of thrombocytopenia was made. And given the patient's clinical and laboratory features that met some criteria for the possibility of TTP, another serious condition with significant mortality, a peripheral blood smear was done to evaluate this differential. The peripheral blood smear, however, did not reveal schistocytes, and further evaluation of the hyperbilirubinemia revealed direct bilirubinemia. With these findings, the diagnosis of TTP was less likely.

Cardiac imaging was requested as one of the initial investigations on presentation. Unfortunately, it was not done then, as the patient presented overnight when echocardiographic services would have been unavailable unless deemed emergent. TTE was to be done at the earliest availability the following day. While awaiting TTE, preliminary blood cultures were noted to be positive for *Staphylococcus *species. With these results, the differential of IE was considered more likely.

*Staphylococcus* bacteremia, whether due to MSSA or methicillin-resistant *S. aureus* (MRSA), poses a high risk of IE. When a diagnosis of *S. aureus* bacteremia is made, cardiac imaging to evaluate for features of IE is recommended. TTE is recommended as the initial test, and in cases similar to ours, where TTE cannot convincingly rule out IE, TEE should be done for confirmation. TEE has a sensitivity of up to 95% for identifying valvular vegetation and has the benefit of identifying associated valvular complications of IE, such as abscesses, dehiscence, and perforation [9]. In our case, TEE confirmed the diagnosis of IE by showing large mobile vegetations on both the anterior and posterior mitral valve leaflets.

The management of IE requires a prolonged course of antibiotics. In addition to antibiotic therapy, surgical intervention is required in about half the cases of IE and has been shown to improve clinical outcomes in specific cases. Current indications for early surgical intervention in cases of IE include (1) persistent fever and bacteremia for at least 7-10 days despite appropriate antibiotics at optimal doses, (2) IE with associated abscess formation or extension surrounding cardiac tissue, (3) IE associated with severe heart failure, (4) large vegetations usually >10 mm in diameter with associated embolic phenomena (e.g., renal or splenic infarcts, stroke), and (5) large vegetations >15 mm even without any clinical evidence of an embolic event due to the significantly high risk of embolic events [5]. With large vegetations of size >10 mm on both mitral valve leaflets and evidence of embolic phenomena, our patient met the criteria for possible surgical intervention. An urgent surgical consult was made.

The timing of surgical intervention has been of much debate over the past years. Early surgical intervention is described as surgery done during hospitalization, prior to the completion of the antibiotic course. Kang et al. have shown that in patients with large vegetations, early surgery significantly reduced mortality and embolic phenomena [10]. In contrast, in other studies, such as that by Kousa et al., early surgery did not affect in-hospital mortality but was associated with a reduction in the length of hospital stay [11].

Another factor often contributing to the optimal timing for surgical management in IE is the development of neurological complications (NCs) such as strokes. Neurologic complications are common complications of IE affecting the left-sided heart valves: the mitral and aortic valves. NCs in IE have a mortality of 21%-83% [12]. Both symptomatic and asymptomatic NCs can occur in IE. Asymptomatic neurological complications are more common than symptomatic neurological complications and occur in about 60%-80% of patients with IE [9].

Neurological complications in IE can be classified into ischemic and hemorrhagic. Ischemic complications occur when emboli arise from the valvular vegetation and occlude vessels of the cerebral circulation. In contrast, hemorrhagic complications occur as a rupture of infective intracerebral aneurysms or as secondary hemorrhage following an ischemic event. Regardless of the underlying pathophysiology, neurologic events can significantly impact the timing of planned surgical procedures in IE. Some literature suggests that surgical intervention within the first two weeks of embolic cerebral infarction is associated with a very low rate of further neurological decline or complications [13,14]. On the other hand, other studies suggest delaying surgery to allow for neurologic recovery as there is a potential risk of worsening ischemic neurologic complications during the cardiac bypass portion of surgical intervention and a potential for worsening the hemorrhagic neurological complications due to anticoagulation with heparin during the procedure [15]. The optimal timing of surgical management in IE should be made on a case-by-case basis, considering the potential risks versus benefits. Most agree that early surgical intervention may be more beneficial in patients with life-threatening complications of IE requiring urgent surgery, even in patients with severe neurological deficits. On the other hand, delaying surgery in cases deemed non-emergent may be more beneficial, particularly in patients with hemorrhagic infarcts.

Our patient developed acute neurological symptoms on the day of planned mitral valve replacement. A neurological examination at that time was significant for mild left hemiparesis only; however, an MRI scan of the brain demonstrated multiple infarcts in the bilateral cerebral hemispheres and the cerebellum. These findings suggest that some cerebral infarcts occurred without overt clinical symptoms or physical examination findings, that is, some infarcts were likely asymptomatic. Moreover, in this case, the patient presented to the ED on this occasion with altered mental status, and head CT done at that time showed no infarcts or hemorrhages. As ischemic infarcts are not often visualized on the head CT until about 24-48 hours, it is possible that the patient's presentation with altered mental status may have been related to acute ischemic cerebral infarcts.

A multidisciplinary discussion of the benefits and risks of early versus late surgical interventions with the patient and his family led to a decision to delay surgical intervention for two weeks, with plans for mitral valve replacement at that time. The patient, however, preferred to have mitral valve repair instead of replacement and, as per his request, was transferred to another hospital out of state.

## Conclusions

Here we have presented a case of IE with an unfortunate delay in diagnosis due to multiple factors, including failure to confidently recognize well-known, but rare peripheral stigmata of IE, and a delay in obtaining cardiac imaging. Our case highlights the need for a high index of clinical suspicion and the recognition of peripheral stigmata of IE to ensure prompt diagnosis and timely initiation of management to decrease morbidity and mortality in these cases.

Another interesting question arising from this case was about the optimal timing of surgical intervention in patients who develop neurologic complications secondary to IE, a question of much debate in the literature. Until there is a consensus on the optimal timing of valvular surgery in these cases, we suggest that the most optimal approach should include a multidisciplinary discussion on the benefits and risks of early versus late (conventional) surgical interventions between the specialists involved in a patient's care and the patient.
